# Virtual online consultations: advantages and limitations (VOCAL) study

**DOI:** 10.1136/bmjopen-2015-009388

**Published:** 2016-01-29

**Authors:** Trisha Greenhalgh, Shanti Vijayaraghavan, Joe Wherton, Sara Shaw, Emma Byrne, Desirée Campbell-Richards, Satya Bhattacharya, Philippa Hanson, Seendy Ramoutar, Charles Gutteridge, Isabel Hodkinson, Anna Collard, Joanne Morris

**Affiliations:** 1 Nuffield Department of Primary Care Health Sciences, University of Oxford, Oxford, UK; 2 Barts Health NHS Trust, London, UK; 3 Blizard Institute, Barts and the London School of Medicine and Dentistry, London, UK; 4 Tower Hamlets Clinical Commissioning Group, London, UK

## Abstract

**Introduction:**

Remote video consultations between clinician and patient are technically possible and increasingly acceptable. They are being introduced in some settings alongside (and occasionally replacing) face-to-face or telephone consultations.

**Methods:**

To explore the advantages and limitations of video consultations, we will conduct in-depth qualitative studies of real consultations (microlevel) embedded in an organisational case study (mesolevel), taking account of national context (macrolevel). The study is based in 2 contrasting clinical settings (diabetes and cancer) in a National Health Service (NHS) acute trust in London, UK. Main data sources are: microlevel—audio, video and screen capture to produce rich multimodal data on 45 remote consultations; mesolevel—interviews, ethnographic observations and analysis of documents within the trust; macrolevel—key informant interviews of national-level stakeholders and document analysis. Data will be analysed and synthesised using a sociotechnical framework developed from structuration theory.

**Ethics approval:**

City Road and Hampstead NHS Research Ethics Committee, 9 December 2014, reference 14/LO/1883.

**Planned outputs:**

We plan outputs for 5 main audiences: (1) academics: research publications and conference presentations; (2) service providers: standard operating procedures, provisional operational guidance and key safety issues; (3) professional bodies and defence societies: summary of relevant findings to inform guidance to members; (4) policymakers: summary of key findings; (5) patients and carers: ‘what to expect in your virtual consultation’.

**Discussion:**

The research literature on video consultations is sparse. Such consultations offer potential advantages to patients (who are spared the cost and inconvenience of travel) and the healthcare system (eg, they may be more cost-effective), but fears have been expressed that they may be clinically risky and/or less acceptable to patients or staff, and they bring significant technical, logistical and regulatory challenges. We anticipate that this study will contribute to a balanced assessment of when, how and in what circumstances this model might be introduced.

**Strengths and limitations of this study**

- To our knowledge, the first major study of remote video consultations from a sociomaterial perspective.
- Aims to collect rich qualitative data that will go beyond ‘technology on versus technology off’ comparisons and illuminate strengths and limitations of this medium in different settings.
- Includes an analysis of the organisational and policy context.
- Not designed to generate an ‘effect size’ or a cost-effectiveness analysis.

## Introduction

### Background

One of the greatest opportunities of the 21st century is the potential to safely harness the power of the technology revolution…to meet the challenges of improving health and providing better, safer, sustainable care for all.—UK National Information Board, November 2014, page 6^1^

Technology-supported consulting is viewed by many as at least a partial solution to the complex challenges of delivering healthcare to an ageing and increasingly diverse population. The health service faces rising rates of chronic illness and dependency, but also a proportion of citizens who are confident to self-manage illness, and improved long-term outlook for serious conditions such as cancer. The UK's National Information Board has argued that to respond effectively to these demographic and epidemiological trends, we need a different kind of health service in which the traditional outpatient consultation, for example, will become increasingly obsolete.^1^

Remote consultations offer potential advantages to patients (who are spared the cost and inconvenience of travel) and the healthcare system (eg, they may be more cost-effective). But fears have been expressed that they may be clinically risky and/or less acceptable to patients or staff, and they bring significant technical, logistical and regulatory challenges.

The evidence base on remote consultations by video technology such as Skype is currently sparse but has begun to accumulate.^2–4^ In particular, a recent review identified 27 published studies of the use of Skype in clinical care, all but one of which reported positive benefits.^4^ Most of these studies were brief descriptions of small, pilot-stage projects (some with as few as five patients). Below, we review the higher quality primary studies from Arnfield and colleague's review that are relevant to our own study along with some additional studies published recently.

A study of family-based behavioural support for adolescents with poorly controlled type 1 diabetes mellitus focused on the ‘working alliance’—that is, the strength of the working relationship between patients, caregivers and healthcare professionals.^5^ The authors found that 10 sessions delivered via Skype were as effective as 10 face-to-face sessions at maintaining the working alliance.^6^ Adherence to treatment and glycaemic control were also similar in the Skype and face-to-face groups.^7^ However, losses to follow-up were high: of 47 (of 92) participants randomised to Skype, follow-up data were available in only 32.

Some studies have studied the use of Skype in the management of other chronic diseases. In one study of the management of depression in older housebound adults, participants were randomised to receive either in-person problem-solving therapy, Skype-delivered problem-solving therapy or a weekly telephone call with no therapeutic content.^8^ Both the in-person and Skype-delivered problem-solving therapy were effective at reducing depression scores and disability outcomes. However, at 36-week follow-up, the participants in the Skype arm of the trial experienced significantly better outcomes than those in the in-person condition. The authors speculated that the more focused nature of the Skype-delivered sessions may have been responsible for these sustained benefits. A study of increased social contact among older adults with access to Skype suggests an alternative explanation: Skype itself may be a valuable tool for wider social integration, thus improving mental health.^9^

A 2014 study reported the use of Skype for orthopaedic clinical follow-up.^10^ The Skype service was offered to 78 patients following total joint arthroplasty. Participants were invited to communicate with their surgeon via Skype, in addition to their scheduled follow-up appointments, on five separate occasions: 1, 3, 4, 6 and 9 weeks. The authors found that 34 of the 78 underwent at least one Skype consultation, whereas 44 did not have appropriate electronic devices or internet connection to use the Skype service. There was no significant difference in clinical outcomes for the users and non-users of this service, though the study was probably underpowered to detect one. However, those followed up by Skype had fewer unscheduled in-clinic visits or called the office for medical advice. Those who had had a Skype consultation rated their postoperative satisfaction as higher than those who had not. In a follow-on paper on 228 participants that encompassed the original sample, the authors found that time spent on the consultation and patient-borne costs were lower in the Skype group.^11^ A linked economic evaluation showed that service costs were also significantly lower in the Skype group.^12^ No patient had an ‘issue missed’, but an accompanying commentary raised the possibility that remote assessment might be less safe.^13^

Virtual clinics via Skype have been used for counselling and mental health consultations. Skype proved an effective medium for supporting independence and self-confidence among young people aged 12–18 years with spina bifida.^14^ In a 15 min consultation once a week, the nurse supported participating patients to improve their continence and self-care. Participants reported that they felt more confident talking about personal issues via Skype rather than face-to-face. They also valued the privacy that the Skype consultations allowed: “I feel more confident, speaking to a nurse on my own about personal things, without my mum being present.” Skype also made it easier for these patients with complex physical needs to ‘attend’ sessions.

In another uncontrolled feasibility study in mental health, Skype consultations were found to be acceptable and feasible in the management of social anxiety disorder in 24 participants who each received 12 weekly sessions of behavioural therapy.^15^ Significant improvements were shown in social anxiety, depression, disability, quality of life and experiential avoidance compared with pretreatment scores.

In a randomised trial of Skype versus standard home care in supporting families with premature infants, the nine families randomised to Skype reported very positive experiences and found the technology easy to use; they specifically commented that video calls were better than ordinary phone calls.^16^ Tellingly, the authors commented, “The families readily embraced the use of ICT, whereas motivating some of the nurses to accept and use ICT was a major challenge” (p.22).

Skype has been used to deliver follow-up training for a technique called ‘pursed lips breathing’ which is used to manage breathlessness in chronic obstructive pulmonary disease. In one small (N=16) study, participants who received the follow-up sessions had better breathlessness management than those with basic training alone;^17^ and another small (N=24) randomised trial confirmed these findings.^18^

A recently published randomised trial compared remote ‘video visits’ in follow-up after surgery for prostate cancer.^19^ Fifty-five men, prescreened for suitability, were randomised to video follow-up or usual care. In this small sample, video visits were assessed as ‘equivalent in efficiency’ to conventional outpatient visits, as measured by amount of time spent face-to-face, patient wait time and total time devoted to care. There were no significant differences in patient perception of visit confidentiality, efficiency, education quality or overall satisfaction. Video visits incurred patient-borne lower costs and were associated with similar levels of urologist satisfaction to conventional outpatient visits.

While this handful of small studies are all broadly positive, the small sample sizes and high losses to follow-up in many studies call into question any unqualified conclusion that the technology is ‘effective’, and the lack of negative studies raises the issue of publication bias. Issues surrounding information governance, informed consent and payment for services, often mentioned in passing in the discussion sections of these studies, are the subject of intense legal and professional debate but have rarely been systematically explored as part of the study protocol.^20^

Technical difficulties are also typically mentioned in passing but not elucidated further. Studies beyond the medical literature have shown that Skype is often ‘laggy’—sometimes the audio and video data are delayed or become unsynchronised. However, in one study that looked at the effect of collaborative song writing as therapy, some participants reported that the lag was actually helpful: it made them pick their words with care and attend more to turn-taking.^21^ There are also times when Skype compresses the video, so that facial expressions are hard to interpret. This is particularly likely when users have other applications running that are using a large amount of bandwidth such as software updates and video streaming.^21^ It may be that the quality of bandwith may be crucial to some (though perhaps not all) kinds of clinical consultation.

We have been working for several years to develop remote consulting as part of business as usual in a busy National Health Service (NHS) trust. Below, we describe the setting and some preliminary data from a set-up phase. We then define the aims, objectives and research questions for the current study, offer a theoretical framework and describe how we will manage the project and collect and analyse the data.

### Setting and context

Barts Health, the UK's largest acute trust, was formed in 2012 when three trusts in different boroughs merged. We will study two clinical services on different sites: Diabetes at Newham and Mile End Hospitals and Pancreatic/Liver Cancer at the Royal London Hospital. These sites are located in two adjacent London boroughs (Newham and Tower Hamlets), characterised by high socioeconomic deprivation and ethnic and linguistic diversity. Burden of disease is high. Like many acute trusts, Barts Health is under pressure to deliver services more cost-effectively while responding to rising need and demand.

The Diabetes service has a long tradition of applied research and quality improvement activity aimed at ensuring that services are accessible, culturally congruent and oriented to meeting the needs of the most vulnerable patients (eg, limited English speakers with low health literacy). A key component of this work has been developing strong links with local general practitioners (GPs) and deploying specialist nurses and bilingual health advocates in community outreach roles.

Unusually, a high proportion of patients with diabetes in this catchment area are young. Newham has one of the youngest populations in the UK and the UK's highest prevalence of type 2 diabetes in the 16–25-year age group (0.57/1000), due to a combination of risk factors (eg, poverty, ethnicity, diet, low exercise levels). Engagement with traditional health service models is low in this demographic, with poor health outcomes (eg, young adults with poorly controlled diabetes have increased risk of sight-threatening retinopathy and adverse pregnancy outcome) and increased use of unplanned care through the A&E department. As explained in the next section, outpatient consultations via Skype for patients who choose this option are already an integral part of this service.

The Royal London HPB (Hepato-pancreato-biliary) Cancer service, led by SB, is a tertiary service to which patients often have to travel long distances when unwell. It provides contrasting organisational, demographic and clinical challenges to the diabetes example while also being nested, broadly speaking, in the same ‘meso’ level context. Patients with pancreatic and liver cancer have a very diverse demographic and may live up to 200 miles away. They have in common a life-threatening diagnosis, major surgery and a prolonged postoperative phase in which they have to cope with multiple physical, emotional and practical challenges. This clinical service has just begun to introduce virtual consultations in order to spare selected patients unnecessary travel. We hypothesise that after the initial face-to-face consultation, some aspects of preoperative preparation and postoperative follow-up will be achievable by remote consultation. But cancer is a sensitive area, so we remain open about the benefit–harm balance.

### Preliminary experiences with remote consulting

We introduced virtual consultations for diabetes at Newham in 2011 and developed the operational aspects of the service, supported by grants from NHS Choices and the Health Foundation. A detailed report submitted to the Health Foundation in December 2014^22^ concluded that virtual consultations were popular with both patients (especially young adults) and staff; 480 remote consultations were documented in 104 patients between 2011 and 2014. In patients who chose to use the remote service, it appeared to be associated with increased engagement (overall ‘did not attend’ rates were 13% in patients accepting the Skype option and 28% in those who chose not to use this option, though denominator populations for these figures were self-selecting and hence not strictly comparable), improved glycaemic control (average glycated haemoglobin level preintroduction and postintroduction of remote consulting was 70 and 65 mmol/L, respectively, for those who used the service) and fewer A&E attendances than those not using the remote service (raw data on this were statistically significant, though numbers were small). While these figures are encouraging, patients were not randomised and there were multiple potential confounders, and 45 patients who initially singed up to the remote service subsequently withdrew from it, so a conclusion that remote consulting ‘works’ would be extremely premature.

## Aims, objectives and research questions

### Aim

To define good practice and inform its implementation in relation to clinician–patient consultations via Skype and similar virtual media.

### Objectives

1. At microlevel, to study the clinician–patient interaction in a maximum variety sample of up to 45 remote outpatient consultations in two clinical areas. In particular, to highlight examples of good communicative practice; to identify and characterise examples of suboptimal communicative practice; and to propose approaches for minimising the latter.
2. At mesolevel, to illuminate and explore the sociotechnical microsystem that supports the remote consultation, thereby identifying how organisations can best support the introduction and sustainability of this service model in areas where it proves acceptable and effective.
3. At macrolevel, to build relationships with key stakeholders nationally and identify from their perspective how to overcome policy and legal barriers to the introduction of remote consultations as a regular service option.

### Research questions

1. What defines ‘quality’ in a virtual consultation and what are the barriers to achieving this?
2. How is a successful virtual consultation achieved in an organisation whose processes and systems are mostly oriented to more traditional consultations?
3. What is the national-level context for the introduction of virtual consultations in NHS organisations, and what measures might incentivise and make these easier?

## Methods

### Study design

Multilevel study with microlevel, mesolevel and macrolevel components. At microlevel, we will study interactional dynamics by generating a multimodal data set (audio transcript, video and computer screen capture) on up to 45 remote consultations. Each ‘case’ will comprise a transcript plus video, analysed sociologically in a way that highlights how one party responds to, and shapes the talk and action of, the other—and how technology affects such human interactions. At mesolevel, we will map the administrative and clinical processes that will need to change to embed online consultations, for example, changes to clinical care pathways, potential changes to staff roles, use of traditional outpatient space, information governance, commissioning tariff. At macrolevel, we will interview national policymakers and other key stakeholders to explore barriers, facilitators and incentives to supporting virtual consultations.

### Theoretical/conceptual framework

We will draw on strong structuration theory (SST), developed by Stones^23^ to extend the seminal sociological work of Giddens. SST acknowledges that in today's world, human actors are often members of multiple social systems and are linked together in complex networks that are fluid and changing.^24^ Greenhalgh and Stones^24^ adapted SST to embrace the adoption, implementation and scaling up of new technologies in health settings.

Structuration theory links the macro of the social environment (social structures) with the micro of human action (agency) and considers how this structure-agency relationship changes over time as society becomes ‘modernised’.^25^ Its central tenet is that society (through rules, norms and meaning systems) profoundly influences—though, importantly, does not *determine*—human behaviour and that human behaviour (through the interpretations and active choices made by individuals) can in turn change society as people challenge and extend what is possible and expected. The structure-agency link is mediated through ‘scripts’ (patterns of behaviour and interaction in social settings, including the use or non-use of particular technologies), which gradually change over time.^26^ Scripts link to organisational routines and hence to the routinisation of innovations.^27^

Central to SST is the role of human agency in engaging with technologies, finding meaning in them and applying the capacity to use them.^28^ SST thus offers potential to theorise *human* characteristics such as identity and social role (eg, what it means to be a ‘professional’ and a ‘patient’), interpersonal relationships (eg, the changing nature of the clinician–patient relationship as paternalism gives way to more egalitarian relations), health literacy, situational knowledge (eg, what each party ‘knows’ about the other's expectations of an interaction), and the physical capabilities needed to operate technology.

SST proposes that external social structures (social norms, rules, expectations and so on) are mediated largely through position-practices (defined as a social position and associated identity and practice), together with the network of social relations that recognise and support it (‘position–practice relations’—of which the clinician–patient relationship are good examples).

**Figure 1:**
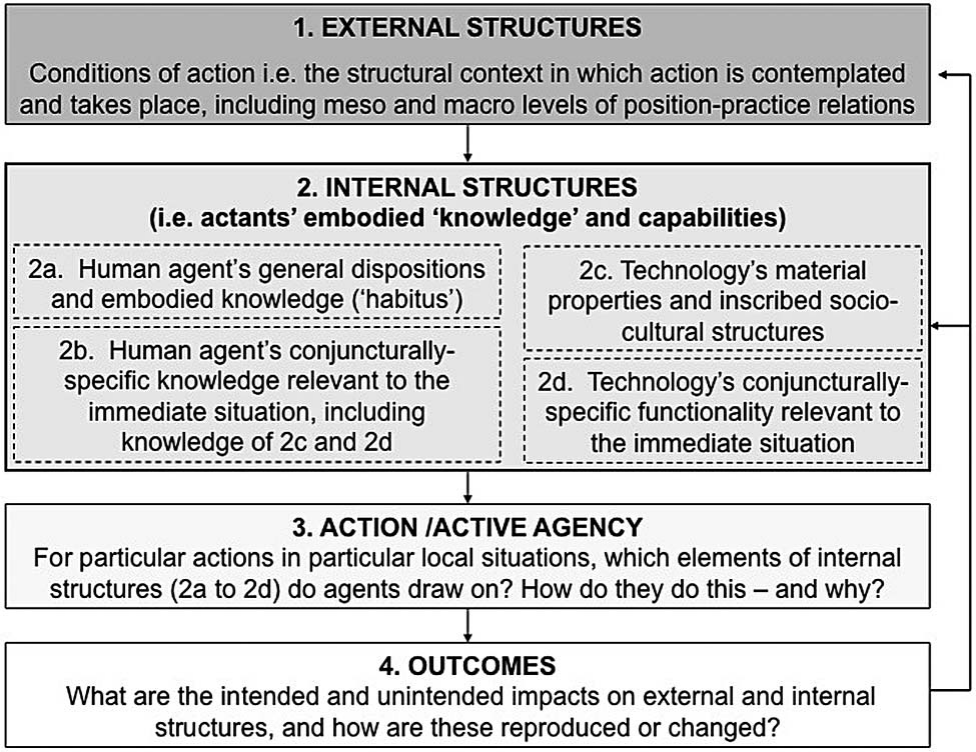
Stones’ strong structuration theory, adapted to encompass a technology dimension (reproduced from Greenhalgh and Stones^24^).

Figure 1 shows the four components of SST that we will study in our analysis of virtual consultations: external structures, internal structures, actions and outcomes. ‘External structures’ refers to the set of position–practice relations referred to above, which are fluid and changing (eg, medicine is, arguably, becoming less paternalistic). Internal structures—the representations of society we carry in our heads—may be divided into:

- *General dispositions*, which include such things as sociocultural schemas, discourses and world views, moral and practical principles, attitudes, ambitions, technical and other embodied skills, and personal values—roughly what Bourdieu^29^ called ‘habitus’; and
- *Particular knowledge* of an aspect of the external world and how one is expected to act within it (eg, a cancer doctor's understanding of what is right and reasonable in a cancer follow-up consultation).

To study actions, we use ethnography to study specific examples of interactions—what Stones calls *conjunctures* (the medical consultation is a good example)—to capture how people play out their position–practice relations, behaving in a way they believe is appropriate and responding in a moment-by-moment way to the other party(ies). To study the agency (ie, human intention) behind these actions, SST incorporates theories from phenomenology (the study of people's shifting fields and horizons of action arising from the focused activity at hand^30^), ethnomethodology (the study of how one person responds, moment-by-moment, to the talk and action of another),^31^ and symbolic interactionism (the study of the subjective meaning and interpretation of human behaviour^32^).

The healthcare setting is heavily institutionalised, and behaviour is often ritualised (ie, we know, and play out, the roles expected of us as doctors, patients and so on). Behaviour in the consultation is strongly influenced by such things as regulations and other governance measures, norms, beliefs, professional and lay codes of practice and deeply held traditions (all of which are embodied and reproduced by human agents including clinicians, administrators and patients) rather than exclusively by business concerns like efficiency and profit. A person's knowledge of these institutional structures (the ‘strategic terrain’ as SST depicts it) may be more or less accurate and more or less adequate. A good example of this might be the older patient who retains the perception that it would be rude to offer suggestions to the doctor, whereas in reality the doctor is keen to promote shared decision-making.

The fourth component of SST (figure 1) is outcomes. The outcome of human action in the consultation may be intended or unintended, and will feed back on both external and internal structures—either preserving them faithfully or changing them as they are enacted. A good example of this in our study is whether a remote consultation that is experienced positively will increase the likelihood that the patient will adhere to treatment and attend (in person or virtually) the next consultation.

In sum, the clinical consultation is a social encounter steeped in moral significance and profoundly influenced by social forces: it is far more than a forum for the exchange of ‘facts’ or the making of ‘decisions’ (shared or otherwise). Clinicians resist technologies which (in their opinion) interfere with good clinical practice and the exercise of professional judgement.^33^ The patient in a clinical encounter will be more or less sick and have socioculturally shaped expectations of being cared for and comforted. Their illness may affect their ability to use the technology (eg, visual or cognitive impairment in diabetes may make the use of computers impossible without help from a carer). An interpreter (lay or professional) may be present. SST provides the potential to turn an analytic lens on how bodily, emotional and cognitive function *interact with* an individual's dispositions, symbolic interpretations and (imperfect) knowledge, possibly interpreted by a third party, to affect how the consultation unfolds. A more extensive exposition of SST in the context of developing and testing e-health technologies is given elsewhere.^34^

The empirical research questions for this study of real-time video consultations in diabetes and cancer are set out above. Expressed in a more theoretical way using the framework of SST, these are:

- How does the dynamic relationship between the macro (external social structures), meso (organisational routines and logics) and micro (individual understandings, dispositions and front-line actions) explain how a real-time video consultation unfolds in the contrasting clinical settings of routine diabetes care and preoperative and postoperative cancer care?
- How do the outcomes of remote video consultations feed back in the short term to change (positively or negatively) position–practice relations of patient and clinician and in the longer term the ability of the organisation and the healthcare system to accommodate and sustain this service model?

### Project management and governance

The study will be delivered via a core working group that meets fortnightly, a six monthly independent steering group and a patient advisory group. The steering group will have a lay chair and cross-sector stakeholder representation (including patients from the patient advisory group and other NHS professionals). The patient advisory group, facilitated by AC (who has a background in community anthropology), will provide advice and feedback to the working group and representatives will attend the wider steering group (supported by AC if required).

Below, we briefly discuss the main ethical issues addressed on our Research Ethics Committee (REC) application form.

The data to be collected for the microlevel study in virtual online consultations—advantages and limitations (VOCAL) are highly personal and sensitive, hence the utmost care must be taken to obtain and maintain informed consent. Consent forms (examples of which are available from the authors) incorporate guidance issued by the General Medical Council on the video-recording of consultations for research purposes, including an opportunity to withdraw consent after the consultation.^35^ The researcher will arrive at the patient's chosen venue (usually their home) at least half an hour before the booked time slot so as to explain the procedure again, confirm consent and get this in writing, and informally discuss the patient's hopes, fears and expectations for the consultation. Another researcher will seek similar consent from the health professional at the clinic base.

Another ethical issue is data management and governance. Videos of consultations are almost impossible to anonymise fully, even with pixilation. We will follow the stringent protocol developed by Swinglehurst^36^ in her doctoral studies. Recordings will be saved directly onto a strictly encrypted portable memory stick and no data of this kind will be placed on networked computers.

### Sampling

The sampling frame includes two clinical groups: diabetes and cancer. In diabetes, we will extend our successful recent pilot study^22^ to a wider group and assess a maximum variety sample, including:

- Young people (16–25 years). Many are busy (eg, at college or work), not well engaged with hospital care, have high ‘did not attend’ rates and risk adverse outcome if lost to follow-up;
- Older people. They may find it difficult to travel because of comorbidity and/or lack of carer;
- Limited English speakers. Some people in this group find the health system difficult to navigate and require an interpreter, who could join the consultation remotely;
- Women who have recently had diabetes in pregnancy. Engagement with the diabetes service (including antenatal care and important postpregnancy reviews, including—for some—an oral glucose tolerance test) may be particularly poor at this time, since many women are busy with young children and/or other duties.^37^

Our sample for cancer is a tertiary care surgical centre in which each patient typically requires multiple contacts, some but not all of which will need a (perhaps lengthy, inconvenient and stressful) trip to an unfamiliar hospital. The following kinds of interaction might suit remote consultations:

- Preliminary orientation. Following a first face-to-face consultation, a nurse might contact the patient remotely to explain what will happen during their hospital admission and deal with questions and concerns;
- Postoperative follow-up. Where clinically appropriate, a convalescing patient with cancer may potentially be seen remotely rather than attend in person;
- Post-treatment surveillance. Patients who have had tests at their local hospital and transmitted to the tertiary centre may be contacted remotely to discuss the results.

Clinician participants will include all consenting members of the clinic teams (senior and junior doctors, specialist nurses). Patient participants will be selected for invitation on the judgement of the clinician, from the denominator population of all those attending participating outpatient clinics. Because remote consulting is a new medium and could potentially have harmful effects in some patients, it is crucial from both a clinical and an ethical perspective that clinicians are able to exercise judgement about which patients to invite to join the study. Exclusion criteria will be: no 3G access at home, lack of familiarity (by patient or family carer) with relevant technology, clinical inappropriateness (eg, need for direct physical examination), inability to give informed consent, and comorbidity preventing participation (eg, severe visual impairment).

The clinic populations include a high proportion of limited English speakers, whose inclusion will be different in different services, reflecting current clinic ways of working. In the young adult diabetes clinic, bilingual health advocates are available and trained in the use of remote consulting, so limited English will not be an exclusion criterion there. In the diabetes antenatal clinic and the cancer clinic, those comfortable with a family member interpreter will be included, but a remote interpreting service will not be available.

We will collect a minimum data set (age, gender, ethnicity, brief reason for exclusion) on patients seen in clinic but not invited to join the study and a similar data set on those who are invited to join it but decline. Training in the use of remote technology, or technical support for its use at home, will not be offered.

The goal of sampling in the microlevel qualitative study is to capture the breadth of experience (of patients and staff) of the remote consultation. We therefore seek a purposive sample of up to 30 diabetes consultations and up to 15 cancer consultations. The lower number in cancer is because there will be far greater practical and ethical challenges to gaining informed consent and avoiding harm, and we do not want to put excessive pressure either on the service as a whole or on individual patients, clinicians or researchers. Within each subsample, and with ethical considerations over-riding (see below), we will seek maximum variety in clinical, social, ethnic and personal circumstances, and in health and IT literacy.

Someone not involved in the study (eg, a receptionist or nurse) will make the initial approach and provide patients with a letter of invitation and consent forms as they arrive for an outpatient consultation. Those wishing to hear more will be contacted by a researcher. A 1-week (minimum) reflection phase will be included to give people time to think about the study before being contacted.

The goal of sampling in the mesolevel study is to map the people, interactions and organisational routines that support the virtual consultation with a view to building a rich ‘ecological’ picture of the sociotechnical microsystem (and its wider embedding in the organisation) needed to make this model work as business-as-usual.^38^ We will begin from the clinic where remote consultations are held, and map the individuals and technologies involved there, then move outwards from this nexus to include estates, finance and clinical informatics departments (among others) in order to explore the organisational change required to embed online care within NHS.

To sample for the national-level interviews, we will begin with individuals charged with delivering IT strategy at NHS England, as well as those leading on patient participation in that organisation, and use snowball sampling (asking each interviewee to nominate a colleague) to build up a picture of the national context. In addition, we will interview key informants at the Royal Colleges (Nursing, Physicians, Surgeons), the National Information Governance Board, Monitor, regulators, professional defence societies and the technology industry. We will ask these national informants to supply us with documents (eg, white papers) which they see as important to guiding emerging policy and practice.

### Data collection: microlevel

The core data set will consist of video-recordings of consultations. The recordings will incorporate two video streams: what the clinician sees and does in the clinic, and what the patient sees and does at the remote site (typically bedroom or living room at home, though sometimes via a hand-held device elsewhere). We will record consultations using a small digital camcorder with wide-angle lens and remote control (eg, Sony Handycam DCR-SR72), mounted on a minitripod and positioned unobtrusively (eg, on a shelf). The camera's field of view will capture as much as possible of the individual and their orientation towards the screen, as well as relevant contextual detail in the room.

We will also capture clinician and patient interaction with the videoconferencing software and other tools used in the consultation. As in previous projects, we will use a commercially available screen capture software tool (ACA Systems) to record screen images showing on each party's computer screen as a video file. This will be run directly from a USB memory stick. The researcher will start and stop the recordings but will leave the room during the consultation. After the consultation, the researcher will confirm that patient and clinician are still willing for the video material to be used in the research.

Each end of the consultation will result in two digital files, one screen capture and one video. Video editing software (Adobe Premier Pro CC) will be used to synchronise the two streams from one end of the consultation into a single editable file.

### Data collection: mesolevel

To map the sociotechnical microsystem that supports the virtual consultation, we will draw on the methodology described by Brown *et al*^38^ ‘Mapping the Sociotechnical Healthcare Ecosystem’, which combines a sociotechnical approach (mapping the people and technologies involved) *and* a human ecology approach (placing particular emphasis on the relationships and interdependencies between these components). Data collection will be predominantly ethnographic, consisting of physically visiting the different departments (clinical, administrative, executive) and undertaking naturalistic interviews—that is, asking people on the job what they are doing and why they are doing it (since, as Barley and Kunda^39^ have shown, people are often unable to talk about the detail of their job unless they are actually doing it at the time), as well as collecting key documentation such as existing standard operating procedures and any informal guides and notes made by staff to help them do their job.

The data set for the meso analysis will thus consist of field notes (to be typed up and annotated as soon as practicable after the field visit), plus documents, charts and other artefacts supplied by staff.

### Data collection: macrolevel

As noted above, one key purpose of the interviews at national level is to build relationships and generate interest in the study with a view to disseminating our findings subsequently. In addition, capturing the perspective of national policymakers is key to a multilevel analysis of the contextual factors accounting for the success and potential transferability of this new service model. To achieve both these ends, we plan a small number of ‘executive-level’ semistructured interviews and collection of relevant policy documents. The provisional interview guide, which will be amended iteratively as findings emerge, is as follows:

- ‘What in your view are the key drivers and facilitators for remote outpatient consultations?’
- ‘How has the policy to promote remote consulting been operationalised in your national organisation so far? What have been its key successes and disappointments? How can you explain each?’
- ‘What do you see as the main challenges nationally to scaling up remote consultations where clinically appropriate?’
- (If not raised spontaneously) ‘What are the information governance challenges to remote consultations? What activity is going on in your national organisation to address these?’
- ‘Which papers or other documents do you think of as guiding policy in this area? What do you think of these documents?’
- ‘Is there anything else I should be asking you or asking other people involved in this project?’

### Data analysis: microlevel

Swinglehurst^36^ developed a detailed methodology for researching clinical consultations with multichannel video. She says (p.86), “The potential of video lies in its ability to access versions of conduct and interaction in everyday settings, explore how talk is inextricably embedded in the material environment and the bodily conduct of participants, and examine the ways in which objects and artefacts come to gain particular significance at particular moments—how material features are invoked, referred to, used, noticed, seen at particular moments for particular purposes.”^40^

Video data are inherently ambiguous. On the one hand, the video record is ‘factual’ and ‘real’—but on the other hand it is not self-interpreting. Indeed, as with a film or play, it is open to multiple different interpretations which will be overlaid by the background and perspective of the viewer.^41^ Video opens up the possibility to combine the analysis of different *modes* such as speech, bodily conduct, gaze and posture. Modes are culturally shaped resources for achieving meaning. A *multimodal* approach is one in which attention is given to all the modes (ie, there is a focus on what is said in parallel with the careful study of ‘body language’). Such multimodal analysis attends to the “complex repertoire of semiotic resources and organizational means that people make meaning through—image, speech, gesture, writing, 3-dimensional forms, and so on” (p.1).^42^ Different aspects of meaning may be expressed by different modes, which may complement each other (or reveal contradictions that can be explored and unpacked).

Following Swinglehurst,^36^ we will apply a qualitative technique, based on discourse analysis, called multimodal linguistic ethnography (which allows the analysis of both talk and actions in context).^43^ ^44^ Such approaches do not offer any specific method that can be applied formulaically. Rather, they provide a number of ‘sensitising concepts’^45^ and tools which can be drawn on in the analytical process.^43^ ^44^ We will adopt an ‘in practice’ perspective, considering how social action is accomplished in and through interaction, and how technology features in this. Central to the analysis will be consideration of the moment-by-moment shaping of interactions, the contingencies that arise when the technology is used in different ways at different times, and how participants orient to these contingencies.

The first step in analysis is transcription, which is an interpretive process involving both immersion in the data and ongoing judgements about what level of detail to include and how to interpret and represent the data (including non-verbal behaviour and body language from both speaker and listener); it is not simply a technical task.^46^ While much of the consultation will be transcribed conventionally (ie, depicted as reported speech), selected sections will benefit from fuller transcription using conventions of conversation analysis (box 1).

Box 1 **Conventions of conversation analysis**

Reproduced with permission from Swinglehurst *et al*,^50^ who in turn draws on Atkinson and Heritage:^98^

**[** onset of overlapping speech; **]** end of spate of overlapping talk

**[[** speakers start a turn simultaneously

**:** preceding sound is lengthened or drawn out (more : means greater prolongation)

**Underlining** emphasis

**(.)** pause of less than 0.2 s; **(0.4)** pause, in 10ths of a second

↑↓ marked rising/falling intonation

**>text<** the talk they surround is quicker than surrounding talk

**°°** the talk they surround is quieter than surrounding talk

**.hhh** inbreath; **Hhh** outbreath

**=**no pause between speakers; contiguous utterances

**(())** a non-verbal activity (eg, the notation ‘C’ might be used to indicate a keystroke)

**(text)** unclear fragment of text 

**.** falling tone (not necessarily end of sentence); ? rising inflection (not necessarily a question)

**CAPITALS** louder than surrounding talk

**<text>** the talk they surround is slower than surrounding talk

We will also apply a more contemporary approach made possible by technical advances in video editing software and work directly with video-recordings. Pearce,^47^ for example, used digital markers (‘tagging’ software) as an aid to analysis so as to engage with his data directly rather than indirectly via a transcript.

Combining these approaches, we will familiarise ourselves with and selectively transcribe the consultations in our data set, adding observations, analytical notes and reflections. We will apply the quadripartite (four-component) framework of SST illustrated in figure 1. For each consultation, we will consider the following:

- External structures (position–practice relations pertinent to this consultation);
- Relevant internal structures of patients and staff (especially what Bourdieu called ‘habitus’—identity, values, internalised codes of practice and particularly clinicians’ perspectives on good clinical practice);
- Material and symbolic properties of the technology and how these shape and constrain interaction;
- Immediate outcomes of the actions and interactions observed.

### Data analysis: mesolevel

In applying Brown *et al*'s framework for analysing the sociotechnical healthcare ecosystem, we will use both diagrams and narrative as synthesising devices to draw together a visual representation and a linked verbal account of the human and technical interactions and interdependencies on which the successful execution of the remote consultation depends. We will also draw on Feldman's^48^ notion of the organisational routine—defined as “a repetitive, recognizable pattern of interdependent actions, involving multiple actors”—whose potential and use in the healthcare setting we have previously described theoretically^49^ and applied empirically.^50^

Routines are how organisational life is patterned. The ethnographic study of routines can illuminate how assimilation of innovations happens (or not). In studying routines for remote consultations, we will identify and compare three things: artefacts such as protocols (Feldman's *proxy routine*); understandings held by staff of how this routine should be enacted (Feldman's *ostensive routine*), arrived at by asking ‘what gets done, by whom, and how?’; and the range of ways in which the routine is actually enacted in an observed instance (Feldman's *performative routine*). We will analyse the convergence and divergence between proxy, ostensive and performative routines to reveal the tension between current business as usual and the new ways of working implied by a remote consultation model.

### Data analysis: macrolevel

Interviews with national stakeholders will be analysed to provide the wider context for understanding what is going on locally. In previous studies of small-scale encounters and organisational routines in healthcare we have found that staff refer (more or less accurately) to such influences as ‘national policy’, ‘National Institute for Health and Care Excellence (NICE) guidance’, ‘the law’, ‘my Royal College’ and ‘information governance law’. Data from direct interviews with national stakeholders, as well as documents recommended or supplied by them (along with their interpretations of these documents) will be compared with statements, actions and interpretations made by organisational actors. In this way, ambiguities will be surfaced and explored, and the key ‘storylines’ that come to summarise complex narratives and shape emerging policy will be identified and explored.^51^ As with the other levels of study, data collection/analysis and theory development will co-evolve.

### Synthesis of data from the different components of the study

Table 1 summarises the data sources and how these will be analysed and synthesised to provide a multilevel case study of the service across two sites.

**Table 1:** Overview of data structure and planned analysis

Data source	Type and nature of data	First-order interpretation	Higher order categories
Descriptive and demographic data on the video consultation service in two settings (diabetes, cancer)	Number of patients offered video option and proportion who accept and persist with it Start and finish time ‘DNA’ (did not attend) rate for video and face-to-face options Unscheduled encounters (eg, A&E) for index condition	Acceptability/popularity of the service Demographic data for example, uptake by age Failed encounter rate Risk of missing serious problems (estimate) Consultation length	Background and context to the multilevel qualitative analysis Could inform economic modelling for future service and/or a future cost-effectiveness study
Microlevel study of 45 clinical consultations (30 diabetes, 15 cancer)	Video recording and screen capture (patient end) Video recording and screen capture (clinician end) Researcher field notes from before/after the consultation, at patient and clinician end	What is said and done in the consultation Unfolding interaction How technology shapes and constrains the consultation How participants felt	External social structures such as Political and economic context Professional standards and definitions of excellence Symbolic meaning of illness Internal social structures (what actors ‘know’ and how they interpret the strategic terrain) ‘Scripts’ held by patients and staff of how they should behave and how they change over time Skills and techniques for using the technology, how these change Assumptions built into technology About capabilities of users About how people interact About privacy and consent Interplay between these factors
Mesolevel study of the sociotechnical microsystem in each setting	People and technologies involved in delivering the virtual consultation Diagrams and accounts of how these relate and interact	Key interactions and interdependencies Key organisational routines and how these are changing over time	
Macrolevel study of wider context for introducing video consulting	Perspective of national stakeholders Documents supplied by these	Historical and policy drivers for the move to virtual consultations	

A&E, accident and emergency.

### The intervention component

Drawing on the principles of action research, we will work with local senior managers and commissioners to understand the organisational change required to embed the remote option. To that end, we will bring staff together six monthly, for a consolidating learning workshop, including gathering feedback from all those involved in, or impacted by, the remote consultation model across all levels of the trust and its linked commissioning GPs.

### Dissemination and projected outputs

This study is investigating a service model that is already being supported and promoted by policymakers despite having (currently) a weak evidence base. We are committed to undertaking a robust study from a position of scientific equipoise. Dissemination is likely to be influenced by whether our study confirms expectations (that this is a worthwhile service model and potentially both practicable and cost-effective in the NHS context) or whether our detailed analysis reveals unexpected disbenefits or even harms associated with it.

A key element of the research design is to draw national policymakers into the study at an early stage via key informant interviews and the macrolevel analysis. These senior policy contacts, who are represented on our steering group, will be strategically placed to implement findings. We will use our multistakeholder steering group to help create widespread interest in the study and appetite for the findings as the research unfolds.

Because there is already political interest in the study, we will be cautious about releasing interim findings but we anticipate being able to use a wide network of colleagues in academia, policy and NHS to disseminate findings once all the data have been analysed and conclusions agreed and signed off by the steering group.

We plan outputs for five main audiences. For academics, we will produce research publications and conference presentations. For service providers, we will propose standard operating procedures, develop provisional operational guidance and highlight key safety issues. For professional bodies and defence societies, we will summarise relevant findings to inform their guidance to members. For policymakers, we will produce succinct and accessible summaries of key findings as relevant to prevailing policy decisions. For patients and carers, our outputs will be distilled into a leaflet and web download, ‘what to expect in your virtual consultation’.

## Discussion

New technologies that support alternatives to face-to-face consulting are seen by policymakers as potentially improving the financial efficiency as well as the clinical effectiveness of services.^52^ As well as video, these technologies include:

- *Telephone*, with various models for assessment and triage of acute problems, with or without clinical advice;^53–66^ GP consultations;^67–69^ call-back services from a doctor to manage heavy demand in general practice, which have been increasingly promoted (see http://www.productiveprimarycare.co.uk/doctor-first.aspx) but to our knowledge not formally evaluated; ‘cold calling’ to offer health education;^70^ and follow-up of chronic illness.^71^ This literature consists mainly of relatively small and heterogeneous primary studies, most of which had significant practical challenges or methodological flaws. Systematic reviewers have tended to conclude that while telephone contact for acute illness may allow minor problems to be dealt with without a face-to-face visit (and sometimes with apparent cost savings), it may miss rare but serious conditions and/or lead to higher rates of face-to-face visits in subsequent days—perhaps because even when patients have been adequately assessed, they may be inadequately reassured. This is particularly the case when call handlers with limited training are working largely to algorithm, as in NHS111.^58^ Telephone consulting, it seems, requires considerable skill and judgement, perhaps because of lack of visual cues. Qualitative studies using conversation analysis have found that compared with traditional face-to-face consulting, telephone consultations have a more linear format and tend to focus on a narrow range of preplanned themes, with less opportunity for the patient to raise issues spontaneously.^67^ ^68^ These rich qualitative findings raise the interesting question of whether the same would be true of video consultations—or whether the addition of high-quality visual medium would emulate the ethos of the face-to-face environment.
- *Text messaging*, for example, for supporting young people with chronic illness;^72^ conveying results of tests^73^ or sending health promotion messages.^74^ ^75^ These studies (which were undertaken on population samples that may not be representative) showed that the text-messaging medium was popular with patients, who used it proactively to send questions (an unanticipated finding) as well as passively (as anticipated) to receive messages sent by health professionals.
- *Email* consultations.^76–78^ Systematic reviews of a large number of primary studies (mostly of weak methodological quality) have confirmed proof of concept (ie, it is *technically possible* to consult via email) and that some sectors of the population *desire* such contact, but have also raised the possibility of increased inequality of access (the service is likely to be used most by young middle class patients, potentially increasing inequality of access for those who are older, poorer and with lower health literacy). Qualitative studies have highlighted professional uncertainty about safety, workload and remuneration, and about the ‘rules of engagement’ for online interaction.^78^
- *Online portals* for prescription ordering,^50^ appointment booking^33^ ^79^ and patient access to their online record.^80^ While these and other research studies have demonstrated proof of concept, such portals are not widely used by patients outside the research setting.
- *Telemedicine*, in which one part of a health service, usually in primary care, links remotely to another, usually in secondary care (eg, telepsychiatry or teleradiology). There are many proof of concept studies^81–85^ and examples of up-and-running services, mostly in remote regions (eg, Scotland http://www.sctt.scot.nhs.uk and Australia http://www.telemedicineaustralia.com.au). But the adoption, spread and sustainability of telemedicine services is often disappointing for complex reasons, including cost, logistics and subtle adverse impacts on professional roles, interactions and work routines.^81^ ^86^
- *Telehealth*, based in the patient's home, in which data on biometric variables (such as blood pressure or oxygen levels) are sent to a data processing centre and (sometime later) evaluated by a health professional who contacts the patent if needed by email or telephone;^87–90^ and *telecare*, in which sensors carried by a person or installed in the home allow remote monitoring of position and/or detect smoke or flooding.^87^ ^88^ ^91–93^ Also known as ‘assisted living technologies’, telehealth and telecare are the subject of much debate. On the one hand, proof of concept (that the technology ‘works’) has been shown for many such technologies and some randomised trials have demonstrated improved outcomes such as reduced hospital admission and mortality rate.^89^ But many trials have been criticised as small, unrepresentative and methodologically flawed, and the largest and best-designed trial achieved improvements in outcomes only at a cost that is probably unaffordable in NHS practice.^89^
- *Combinations of the above*—for example, a systematic review of the cost-effectiveness of ‘telehealth’ that included both home-based and telemedicine services, which showed that both the efficacy and costs of such services varied considerably across studies.^90^

The contribution of these technologies to healthcare has been studied mainly using experimental methods (especially randomised controlled trials). Much of the literature reviewed above is *technology-focused*—classifying service models primarily by the nature of the technology and secondarily by the task supported by that technology. Elsewhere, we have criticised technology-focused experimental research, arguing that comparing ‘technology-mediated care’ with ‘usual care’ using a set of predefined outcome measures is a crude and deterministic approach to a complex topic.^87^ ^94–97^

While experimental studies have their place, they are not the design of choice for teasing out the (often subtle) social and material interactions occurring between patient, staff member and technology(ies). Only in-depth qualitative studies can reveal how technology's material properties and affordances interact with users’ identities, experiences, expectations and capabilities to shape and constrain interactions. Barley^26^ has called the introduction of a new technology in healthcare ‘an occasion for structuring’. In other words, the introduction of the video medium offers possibilities for clinicians and patients to start to interact differently, potentially making the consultation more—or less—efficient, effective and patient-centred.

As noted in the introduction, the research literature on remote video consultations is sparse. Not only have there been no robust qualitative studies of the kind we plan in the VOCAL project, there are no adequately powered randomised trials and few controlled before-and-after studies. The few studies conducted to date have shown great potential for the use of virtual online media tools, such as Skype, for video-based communication between patient and clinician. These studies have generally focused on evaluating the outcomes of the technology intervention (eg, clinical biomarkers, service utilisation).

While these insights are important, we must also understand the complex and inter-related challenges that teams will face—at both local and national level—when attempting to embed the technology within healthcare organisations. Our unique multilevel analytic approach will, we hope, illuminate the complexity of the remote video consultation and the system in which it is nested (including organisational, legal, regulatory and policy contexts), thereby contributing to a balanced, theory-based assessment of when, how and in what circumstances this service model might be introduced.
